# Empathy in senior year and first year medical students: a cross-sectional study

**DOI:** 10.1186/1472-6920-11-52

**Published:** 2011-07-29

**Authors:** Eunice Magalhães, Ana P Salgueira, Patrício Costa, Manuel J Costa

**Affiliations:** 1 School of Health Sciences, University of Minho, Braga, Portugal

## Abstract

**Background:**

The importance of fostering the development of empathy in undergraduate students is continuously emphasized in international recommendations for medical education. Paradoxically, some studies in the North-American context using self-reported measures have found that empathy declines during undergraduate medical training. Empathy is also known to be gender dependent- (highest for female medical students) and related to specialty preference - (higher in patient-oriented than technology-oriented specialties). This factor has not been studied in Portuguese medical schools.

**Methods:**

This is a cross-sectional study of undergraduate medical students on self-rated measures of empathy collected at entrance and at the conclusion of the medical degree, and on the association of empathy measures with gender and specialty preferences in one medical school in Portugal. Empathy was assessed using the Portuguese adaptation of the Jefferson Scale of Physician Empathy-students version (JSPE-spv) among three cohorts of undergraduate medical students in the first (N = 356) and last (N = 120) year. The construct validity of JSPE-spv was cross-validated with Principal Component Analysis and Confirmatory Factor Analysis. Reliability was assessed using Cronbach' Alpha. Global JSPE-spv score differences were examined by year of medical school, gender and specialty preferences (people-oriented vs technology-oriented specialties).

**Results:**

The empathy scores of students in the final year were higher as compared to first year students (F (1,387) = 19.33, p < .001, ɳ^2^_p _= 0.48; π = 0.99). Female students had higher empathy scores than male students (F (1,387) = 8.82, p < .01, ɳ ^2^_p _= 0.23; π = 0.84). Significant differences in empathy were not found between the students who prefer people-oriented specialties compared to those who favor the technology-oriented specialties (F (1,387) = 2.44, p = .12, ɳ ^2^_p _= 0.06; π = 0.06).

**Conclusions:**

This cross-sectional study in one medical school in Portugal showed that the empathy measures of senior year students were higher than the scores of freshmen. A longitudinal cohort study is needed to test variations in students' empathy measures throughout medical school.

## Background

Physicians who are able to establish good relationships with patients achieve better compliance [1], better patient satisfaction [1,2] and better clinical outcomes [3]. Empathy is one of the most influential "ingredients" of good physician-patient relationships [4]. A recent review defines empathy succinctly as the "appropriate understanding of the patient" [5]. The definition of empathy in the context of patient care used in this work was advanced by Hojat (2007) as a "predominantly cognitive (rather than an emotional) attribute that involves an understanding (rather than feeling) of the patient's experiences, concerns, and perspectives of the patient, combined with a capacity to communicate this understanding" [[4], p.80].

Empathy has been characterized in distinct ways in the medical education literature - from a personality trait [6] to a cognitive attribute [5] - but the view that empathy includes a cognitive component is consensual, i.e., one that refers to the ability of physicians to understand patients' emotions and to communicate such understanding [7]. Such a cognitive component should be amenable to training and, thus, medical schools can play a positive role in the development of students' understanding about empathy [8].

Despite a general awareness of the importance of physician empathy in patient care, some studies in the North-American context have found a decline in self-reported measures of empathy of undergraduate students throughout medical school [8-10] and post-graduate training [11]. In those studies it is suggested that "erosions" in empathy can be associated with the learning context, the "hidden curriculum", student difficulties in dealing with stressors in medical education, and poor role modelling in the academic and clinical workplaces [12,13]. The disturbing possibility is that medical education might be injuring instead of nurturing empathy. Most of the evidence for a decline in empathy originates from studies developed in medical schools in the USA [8-10]. There is only one study outside the USA conducted in Trinidad and Tobago that shows a decrease of self-reported empathy [14]. The generalization of findings within the USA or elsewhere is uncertain, since the studies were restricted to one medical school and were based on self-reported measures of empathy - usually derived from physician scores on instruments completed in the absence of patients. Recent cross-sectional studies in Japan and Korea found the highest values for measures of empathy, by year of medical school, in senior students [15,16]. A cross-sectional study in Iran did not find variations in empathy [17]. The effect of undergraduate medical training on the development of medical students' empathy remains unclear.

There are research instruments available to measure the multifaceted construct of empathy. Among the self-reported instruments applicable in the context of medical education (e.g., Interpersonal Reactivity Index, Balanced Emotional Empathy Scale) [4-6,18], the Jefferson Scale of Physician Empathy (JSPE) is specific to patient care and exists in two versions, the physician version and the student version, both of which have been submitted to psychometric evaluation. The face validity, construct and content validity, criterion-related validity, and reliability of the scale have been demonstrated for the original English version in the USA [11,19]. The student version of JSPE has been adapted to several countries and languages [11,15-17,19-21] including Portugal [22]. Although the JSPE student version assesses the students' orientation towards empathy, JSPE measures have been found to be associated with behaviours of empathy [4].

### Purpose of the study

As part of an ongoing longitudinal study with multiple cohorts, medical students in the School of Health Sciences of the University of Minho in Braga, Portugal were asked to complete the Portuguese adaptation of the JSPE (JSPE-spv) [22]. The present cross-sectional analysis addresses the differences in empathy scores between first year and senior students, between genders, and between specialty preferences. The research hypotheses were that empathy scores for first year medical students will be higher than for senior students, the scores for female students will be higher than the scores for male students' scores and a student preference for "people-oriented" specialties is associated with higher empathy scores as compared with a preference for "technology-oriented" specialties.

## Methods

### Participants

Participants included 476 medical students from 6 entering classes at the School of Health Sciences - University of Minho, in the first (N = 356) and sixth year (N = 120) of the curriculum. There were 321 females (67.4%) and 155 males (32.6%) students in the study population.

Three cohorts completed the questionnaires in the 1st year (cohorts defined here as 4, 5, 6) and 3 cohorts in the 6th year (cohorts defined here as 1, 2, 3). The study sample includes all students for whom the complete sets of data were available. The data were extracted from University of Minho's Medical Education Unit longitudinal database, which was the central repository for individual student data. Responses from first year medical students were collected at the beginning of the medical school and the responses from sixth year students at the end of training. The curriculum and the teaching methods were stable over the period in which the two cohorts were assessed. The response rate for the total sample was 92% (Table 1).

**Table 1:** Description of study participants

		Frequency (%)	Response rate* (%)
Academic Year	1^st ^year	356 (74,7)	
	6^th ^year	120 (25,3)	
Gender	Females	321 (67,4)	
	Males	155 (32,6)	
Cohort			
(year of entering in medical school)	1 (2001)	43 (9)	86
	2 (2002)	30 (6.3)	79
	3 (2003)	47 (9.9)	94
	4 (2007)	105 (22.1)	95
	5 (2008)	130 (27.3)	94
	6 (2009)	121 (25.4)	93
	Total	476 (100)	92

### Instruments

The medical students completed two questionnaires: the Jefferson Scale of Physician Empathy - students' Portuguese version (JSPE-spv) and an Admission Survey developed locally that includes an item asking students about their specialty preferences at the time.

#### Jefferson Scale of Physician Empathy (JSPE) - students Portuguese version

The JSPE-spv includes 20 Likert scale items which are scored from 1 (Strongly disagree) to 7 (strongly agree). The 20 items are classified according to one of three subscales: *"Perspective Taking" *(10 items); *"Compassionate Care*" (8 items) and *"Standing in the Patient's Shoes*" (2 items). The translation and adaptation of JSPE-sv has been described in a Portuguese publication [22] and followed established research guidelines [23]. The JSPE-spv was translated into Portuguese by a researcher with a detailed understanding of the instrument. Subsequently the instrument was reviewed by two bilingual individuals, and the "*Modified Direct Translation*" method was applied [23]. The back-translation was conducted by a native Portuguese speaker fluent in English. The latter version was then sent to the authors of the original version for their approval. The psychometric properties of JSPE-spv were previously tested with a different sample with a confirmatory factor analysis approach [22].

#### Specialty Preferences

This study focused on the following item of the Admission Survey: "*What is the specialty that you might consider choosing in the future?" *Forty-seven possible specialties choices were listed in this item. Student preferences were classified into two previously defined broad groups designated as "people-oriented" and "technology-oriented" specialties [21]. The "people-oriented" specialties require extensive encounters with patients and attention to psychosocial factors (e.g., Primary Care, Gynecology/Obstetrics, Psychiatry, Pediatrics, Internal Medicine and Cardiology). The "technology-oriented" specialties are centered on procedurals and require technical skills (e.g., Anesthesiology, General Surgery, Orthopedics and Radiology) [4].

### Procedures

Participation was voluntarily and students were assured that their responses were confidentiality. Informed consent was obtained from all participants. Evidence of construct validity of scores was collected with the present sample and cross-validated. Data were analysed with PASW Statistics 18 (Predictive Analytics SoftWare Statistics) [24] and AMOS 18 [25].

### Statistical Analyses

Two-way ANOVA was computed to assess differences on total scores related to gender, specialty preferences and year of medical school (first year vs. sixth year), and MANOVA was used to assess differences on the three dimensions of empathy. The absolute values of skewness and kurtosis for all items were within the acceptable range of the normal distribution (lower than 3.0 and 8.0, respectively) [26]. The cross-validation of the JSPE-spv structure was assessed using a holdout method with Principal Component Analysis and Confirmatory Factor Analysis, applied to two sub-samples which included 238 participants each (A and B) obtained from randomization of the full sample. Sub-sample A was subjected to an exploratory principal component analysis with *Varimax *rotation. The fit of the exploratory structure retained in this first step was then assessed to sample B using confirmatory factor analysis with Maximum Likelihood estimation. Reliability was estimated using *Cronbach *Alpha.

## Results

### Retest the construct validity of JSPE-spv

To strengthen the findings regarding differences in empathy measures as a function of medical training, we retested the psychometric characteristics of the instrument with the present sample. A previous exploratory study tested the factorial structure without a holdout method of cross-validation [22]. The present study follows a cross-validation process with Principal Components Analysis (PCA).

Our tests of the necessary assumptions to the application of PCA were successful: KMO = 0.77 (i.e., measure of sampling adequacy test) and Bartlett's Test of Sphericity was significant (p < .001) (i.e., the test of significance of correlation between variables). The cross-validation revealed a factorial structure that was in accordance with the three dimensions of original version, with the exception of six items that showed the highest loadings on unintended components (2, 10, 13, 18, 19, and 20) and two items (18,19) that showed poor loadings (lower than .30) (cf. Table 2).

**Table 2:** Principal Components with Varimax rotation solutions of JSPE-vs items

Item	Communalities		Components		Correlation
		Compassionate care	Perspective taking	Standing in the Patient's Shoes	r***
14. I believe that emotion has no place in the treatment of medical illness	.488	**.694**	-.016	.073	.594
8. Attentiveness to patients' personal experiences does not influence treatment outcomes	.466	**.662**	.089	.140	.591
1. Physicians' understanding of their patients' feelings and the feeling of their patients' families does not influence medical or surgical treatment	.423	**.624**	-.179	.030	.412
20. I believe that empathy is an important therapeutic factor in medical treatment	.512	**.583**	.411	-.054	.608
10. Patients value a physician's understanding of their feelings which is therapeutic in its own right	.376	**.572**	.219	.017	.553
13. Physicians should try to understand what is going on in their patients' minds by paying attention to their non-verbal cues and body language	.368	**.528**	.274	.117	.524
7. Attention to patients' emotions is not important in history taking	.243	**.469**	.126	-.081	.447
2. Patients feel better when their physicians understand their feelings	.247	**.454**	.170	.111	.346
11. Patients' illnesses can be cured only by medical or surgical treatment; therefore, physicians' emotional ties with their patients do not have a significant influence in medical or surgical treatment	.251	**.444**	.133	.190	.499
12. Asking patients about what is happening in their personal lives is not helpful in understanding their physical complaints.	.230	**.394**	-.015	.273	.466
17. Physicians should try to think like their patients in order to render better care	.520	.005	**.720**	.034	.435
9. Physicians should try to stand in their patients' shoes when providing care to them	.469	.085	**.658**	.167	.499
16. Physicians' understanding of the emotional status of their patients, as well as that of their families is one important component of the physician-patient relationship	.622	.454	**.644**	-.037	.612
15. Empathy is a therapeutic skill without which the physician' s success is limited	.382	.326	**.504**	-.147	.484
5. A physician's sense of humor contributes to a better clinical outcome	.215	.260	**.370**	-.102	.387
4. Understanding body language is as important as verbal communication in physician-patient relationships	.217	.196	**.364**	.214	.338
18. Physicians should not allow themselves to be influenced by strong personal bonds between their patients and their family members	.090	.138	**-.264**	.039	.192
6. Because people are different, it is difficult to see things from patients' perspectives	.690	-.068	.022	**.828**	.248
3. It is a difficult for a physician to view things from patients' perspectives	.565	.101	-.093	**.739**	.298
19. I do not enjoy reading non-medical literature or the arts	.108	.192	.075	**.256**	.216
Eigenvalues		4.42	1.69	1.36	
% of Explained Variance		17.65	11.85	7.89	
Cronbach's Alpha		.63	.74	.64	

The total variance explained by the three dimensions of empathy was 37.4% which is similar to the values reported in the literature [17]. Confirmatory Factor Analysis (CFA) revealed that the model with "no correlated errors" (Fit Model A) displayed poor fit index values, based on the χ2/df ratio, the *Comparative Fit Index *(CFI) and *Root Mean Square Error of Aproximation *(RMSEA) [27,28]. Therefore, a second model was tested, with possible violations of "no correlated errors" (Fit Model B). A satisfactory level of model fit was achieved (Table 3).

**Table 3:** Fit Indices for Empathy model

	**χ^2^(df) Sig**.	**Ratio χ^2^/df**	**TLI**	**CFI**	**RMSEA (HI90)**
	
Model A	481,401 (173) ***	2.8	.57	.61	.087 (.096)
Model B	200,444 (160)*	1.3	.94	.95	.033 (.046)

Cronbach's Alpha for total scale was .77 which is similar to previous reliability values (.76) reported in the Portuguese publication. These values are below those reported by the original in the USA [4], but similar to the results found for adaptations developed in the Republic of Korea and Japanese [15,16].

### Student empathy: comparisons considering the stage of training in medical school, gender and specialty preferences

Our tests of the homogeneity of variances by the Levene' test were succsessful (F(7) = 1.23; p = .287). A comparative analysis of the mean JSPE-vs scores, revealed that measures for seniors (*M *= 118.21; *SD *= 9.10) were statistically higher than for first year students (*M *= 110.31; *SD *= 10.63; F (1,387) = 19.33, p < .001, ɳ ^2^_p _= 0.48; π = 0.99). The self-reported measures showed that students in later stages of training had higher scores on two dimensions of the scale: "Perspective taking" (*M *= 59.38; *SD *= 6.31; F (1,475) = 27.41, p < .001, n^2^_p _= 0.55; π = 0.99) compared to freshmen (*M *= 55.82; *SD *= 6.48) and also for "Compassionate Care" (F (1,475) = 32.31, p < .001, ɳ ^2^_p _= 0.64; π = 1.00; Seniors (*M *= 48.78; *SD *= 4.04) compared to freshmen (*M *= 45.81; *SD *= 5.22). No significant differences were found on the third dimension "*Standing in the Patient's Shoes*".

Therefore, the data contradicted the first hypothesis that the empathy total score of entering students is higher than in seniors and concur with previous cross-sectional studies that found highest measures of empathy in senior medical students [15,16].

In terms of comparisons by gender, the empathy scores of female students (*M *= 112.86; *SD *= 10.81) were higher than the scores of male students (*M *= 110.32; *SD *= 10.69; F (1,387) = 8.82, p < .01, ɳ ^2^_p _= 0.23; π = 0.84). Female students (*M *= 47.17; *SD *= 4.86) scored significantly higher than males merely on "Compassionate Care" (*M *= 45.30; *SD *= 5.38; F (1,475) = 14.53, p < .001, ɳ ^2^_p _= 0.30; π = 0.97). No significant differences were found on "Perspective Taking" and "*Standing in the Patient's Shoes*".

No significant differences were found between students with a preference for "people-oriented" (*M *= 113.18; *SD *= 10.92) vs "technology-oriented" specialties (*M *= 110.77; *SD *= 10.52; F (1,387) = 2.44, p = .12, ɳ^2^_p _= 0.06; π = 0.06).

The Multivariate Analysis of Variance reveals an interaction effect between medical stage of training and specialty preferences, and between gender and medical stage of training. Specifically, the female students in the sixth year (*M *= 120.77; *SD *= 7.46) scored significantly higher on JSPE-spv than male students (*M *= 113.19; *SD *= 10.01; t(118) = -3.98 p < .001), but no statistically significant gender differences were found by gender in first year students. Students who preferred "people-oriented" specialties on the 6^th ^year (*M *= 119.85; *SD *= 8.29) scored significantly higher on JSPE-spv than "technology oriented" students (*M *= 113.84; *SD *= 9.86; t(90) = -2.94 p < .01). No statistically significant differences in empathy scores by specialty preferences were found among 1^st ^year students.

No interaction effects were found between gender and specialty preferences nor between gender, specialty preferences and medical stage of training (cf. Table 4).

**Table 4:** Two way ANOVA: the association of empathy with specialty preferences, gender and Medical stage of training

	F	P-value	**η**^**2**^_**p**_	π
Gender	8.816	.003	.023	.842
Specialty Preferences	2.438	.119	.006	.344
Medical stage of training	19.326	.000	.048	.992
Gender*Specialty Preferences	.004	.953	.000	.050
Gender*Medical stage of training	5.482	.020	.014	.646
Specialty Preferences*Medical stage of training	4.025	.046	.010	.517
Gender*Specialty Preferences* Medical stage of training	1.511	.220	.004	.232

## Discussion

The present cross-sectional study collected measures of empathy using the JSPE-spv from 6 cohorts of undergraduate students, to compare the students' understanding about empathy in seniors and first year medical students. Our findings are similar to those of past studies undertaken with 6 year undergraduate medical programs with Japanese and Korean versions of the instrument [15,16]. Even though no causal interpretations should be made in terms of increases empathy scores due to the cross-sectional design of the study, they open the possibility that the measures might have increased during medical training. To clarify how empathy measures vary throughout undergraduate medical education, an ongoing longitudinal study is collecting repeated measures of empathy of the same cohorts in years one and six.

This study identified differences on JSPE-spv scores by gender, confirming findings from other reports [7,8,21]. The study also found an interaction effect between stage of training and gender as the only significant gender differences in empathy scores were found in 6^th ^year students. We can offer two non-exclusive explanations for the gender differences. One is based on the evolutionary theory of parental investment, according to which females are expected to develop a stronger sense of caring for offspring than men [11], and should thus be more skilled in understanding their offspring and in communicating such understanding. There is a possible parallel between such skills, as applied to offspring, and empathy, as applied to patients. This is consistent with the findings that the gender differences could be traced to the "Compassionate Care" dimension of the scale. The second explanation would be related to differences between genders in role expectations. Females are more likely to develop interpersonal relationships and to offer emotional support than males [11,15,20,21], and tend to exhibit more social sensitivity and humanistic and care-oriented attitudes, whereas men tend to adopt justice-oriented attitudes, dominance, independence and control [7].

The cross-validation of the psychometric properties of the JSPE-spv through Principal Component Analysis with the study sample, replicated the three factors in the Portuguese version original model, "Compassionate Care", "Perspective Taking" and "Standing in the patients shoes", and explained 37% of variance. This is similar to results obtained in previous research [16]. The percentage of variance explained by JSPE-spv is relatively low, nevertheless, according to Hair and colleagues (1998) in the Social Sciences, solutions that account for 60% or even less of the total variance are considered satisfactory [29]. Confirmatory factor analysis modelling of the exploratory solution also yielded a good model fit with item correlated errors. Also, the reliability value of the Portuguese version (Cronbach' Alpha .77), albeit lower than the original (Cronbach' Alpha .89), is above the '.7 value and similar to other versions of JSPE (e.g., the Japanese version with Cronbach' Alpha of 0.80) [15]. As to the two items with poor loadings, they were maintained in the JSPE-spv after verification that their exclusion would lead to a minor improvement of the scale's reliability (Cronbach' Alpha 0.78 if items deleted).

Additionally, to test the influence of such items on our results, an alternative ANOVA was performed considering the dependent variable "JSPE-sv score" computed without those two items and the all conclusions remain [Gender: F(1,380) = 6.77, p < .05; Specialty Preferences: F(1,380) = 3.17, p = .08; Medical stage of training: F(1,380) = 16.07, p < .001]. Maintaining all items of the original JSPE-spv allows comparison with international studies using the same scale.

There are several potential limitations to consider. Firstly, our study is cross-sectional and not a longitudinal follow up. As such it does not reflect a real modelling of growth in empathy scores in the student cohorts. Secondly, the scores reported were derived from measures obtained with a self-reported instrument that have not been complemented with observational measurements.

The higher empathy scores among senior medical students could be cohort effects, but could also reflect the influence of training. It is not known which educational elements might be associated with the latter possibility. One plausible candidate would be the curricular emphasis on the principles of humanism and patient centeredness in medical care. This begins in the four weeks of medical school. A vertically integrated humanities program running from year 1 up to year 6, maintains this emphasis. There are other important elements across the curriculum aimed at nurturing the development of empathy. The training of communication skills starts in the second year. Students interview a family at different points in time during the second and third years. Twenty per cent of the clinical clerkship time spent in primary care in urban, sub-urban and rural settings. Clerkship assessments include the clinical teachers' score of student "professionalism". Each student is assessed up to 25 times during undergraduate studies (i.e., one assessment at each rotation) on this factor.

Further research is needed to identify how the formal curriculum may foster the growth of empathy in medical students [30,31]. Complementary methods and instruments, such as peer assessment or observational approaches, would be valuable contributions to the study of variation in student empathy.

## Conclusions

Our results showed that sixth year students displayed higher scores of empathy than first year medical students. There were significant associations between gender and empathy scores. Our findings also add a third undergraduate medical program to the short list of programs that have reported data on positive cross-sectional self-reported empathy variation during medical school. Results will be confirmed with a longitudinal design, already under way.

## Competing interests

The authors declare that they have no competing interests.

## Authors' contributions

All authors designed the study. EM and AS administered the surveys. EM and PC developed the statistical analysis. EM wrote the first draft of the manuscript. All authors have reviewed and approved the text of the manuscript.

## Pre-publication history

The pre-publication history for this paper can be accessed here:

http://www.biomedcentral.com/1472-6920/11/52/prepub
